# Use of magnetic resonance imaging to determine laterality of meniscal size in healthy volunteers

**DOI:** 10.1371/journal.pone.0228040

**Published:** 2020-01-23

**Authors:** Mohammad Hamdan, Bassem Haddad, Ula Isleem, Rami Yaghi, Salsabiela Bani Hamad, Rahaf Al-Balkhi, Rami Afifi, Saif Aldeen Alryalat, Fadi Hadidi, Aws Khanfar, Amjad Shatarat

**Affiliations:** 1 Jordan University Hospital, Department of Orthopaedic Surgery, Queen Rania Street, Amman, Jordan; 2 University of Jordan, Faculty of Medicine, Queen Rania Street, Amman, Jordan; 3 Jordan University Hospital, Department of Opthalmology, Queen Rania Street, Amman, Jordan; 4 University of Jordan, Department of Anatomy, Queen Rania Street, Amman, Jordan; Rothman Institute, UNITED STATES

## Abstract

**Introduction:**

The menisci are responsible for several functions. They are shock absorbers during dynamic loading on the knee and provide a broader surface area on which to distribute stress evenly to the tibia and femur. These functions allow for smoother movement and greater stability of the knee joint. Meniscal injury can be a great impediment to the function of the knee. Therefore, in the case of meniscal injury, our main concern is the relief of patient symptoms, followed by consequent restoration of meniscal function to the greatest of our ability.

To prevent the long terms effects of a meniscectomy, meniscal allograft transplantation (MAT) was developed. The potential of using the size of the contralateral healthy menisci, to determine the size of the menisci to be replaced, will be discussed.

**Methods:**

Knee MRIs done on healthy patients in the past 5 years were reviewed. Magnetic Resonance Imaging was performed using a 3-T scanner. Each individual was examined with knee joints in full extension. Measurements were performed two separate times, two weeks apart. A mean of three measurements was made during each session to reduce error.

Thirty-eight normal bilateral knee joints MRIs remained (16 males, 22 females). Participants were sampled from the institutional Picture Archiving and Communication System (PACS). Age, gender, and the medial meniscal and lateral meniscal size of both knees were recorded. The laterality of the menisci was compared between both knees in each patient.

**Results:**

A total of 38 patients were included in this study, with a mean age of 37.39 (±9.50) years. They were 16 (42.1%) men and 22 (57.9%) women. We didn’t find any significant difference in the mid-coronal section between left and right knees meniscal measurements. None of the measurements were significantly different between men and women. There was no significant difference in the medial mid-sagittal section or lateral mid-sagittal section between left and right knee meniscal measurements.

**Conclusion:**

The results obtained in this study may support the use of MRI of the bilateral knee to obtain an appropriately sized allograft.

## Introduction

The menisci are crescent-shaped fibrocartilage structures which cover around two-thirds of their corresponding tibial plateau, on the medial and lateral aspects of the knee. The horns of the medial meniscus usually insert into the flat intercondylar region of the tibial plateau anteriorly, and just anterior to the insertion of the posterior cruciate ligament, posteriorly. The anterior horn of the lateral meniscus attaches on the tibia, just posterior and lateral to the anterior cruciate ligament, while the posterior horn attaches between the posterior cruciate ligament and the posterior horn on the medial meniscus [1].

The menisci are responsible for several functions. They are shock absorbers during dynamic loading on the knee and provide a broader surface area on which to distribute stress evenly to the tibia and femur. These functions allow for smoother movement and greater stability of the knee joint [2]. These functions can be attributed to the composition of the menisci, primarily type 1 collagen fiber bundles, which give them their characteristic tensile strength and stiffness [3]. For these reasons, meniscal injury can be a great impediment to the function of the knee. Therefore, in the case of meniscal injury, our main concern is the relief of patient symptoms, followed by consequent restoration of the meniscal function to the greatest of our ability.

In the setting of degenerative or irreparable tears in the meniscus, a total or subtotal meniscectomy may be the only definitive treatment.

To prevent the long terms effects of a meniscectomy, meniscal allograft transplantation (MAT) was developed. A meta-analysis study performed in 2014, showed that all studies describing MAT demonstrated postoperative clinical improvement in patients who underwent this procedure, regardless of the type. It also showed that the most common mode of imaging was radiographic. [4] In this study, bilateral Magnetic Resonance Imaging (MRI) of the knees were used to compare the laterality of the menisci in both knees. The potential of using the size of the contralateral healthy menisci, to determine the size of the menisci to be replaced, will be discussed.

## Materials and methods

After acquiring the approval of the Institutional Review Board (IRB) of the Jordan University Hospital and the University of Jordan-Faculty of Medicine, knee MRIs done on healthy patients in the past 5 years at Jordan University Hospital were reviewed. Informed consent was waived by both IRBs due to the fact that the images did not include identifying patient information. All images were received with anonymization of the patient name and file number. All images followed a standard protocol. Magnetic Resonance Imaging was performed using a 3-T scanner.

Coronal images were taken parallel to the axis of the tibial plateau. For the T2 weighted fat-suppression coronal STIR cuts, the repetition time was 4,330 ms, an echo time of 20.0 ms, a slice thickness of 3.0 mm, and an interslice gap of 0.5mm. Sagittal images were taken perpendicular to the tibial plateau. For the T2 weighted fat suppression sagittal STIR cuts, the repetition time was 4,412 ms, and echo time of 21 ms, a slice thickness of 3.0 mm, and an interslice gap of 0.4 mm. The field of view for both types of cuts was 16*16 cm.

**Fig 1:**
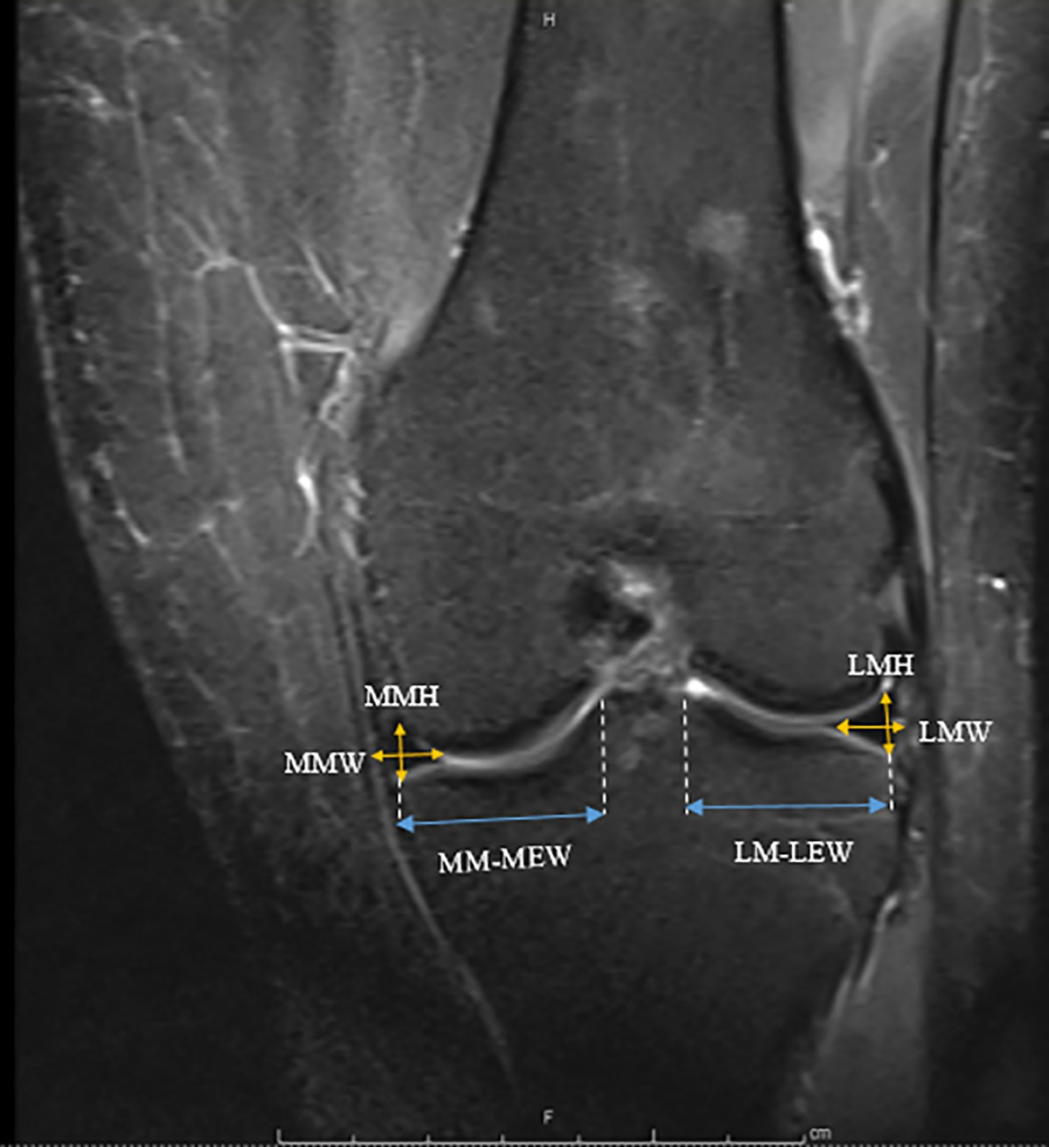
Mid-coronal section (MMH) Medial Meniscal Height, (MMW) Medial Meniscal Width (MM-MEW) MM-Medial Eminence Width (LMH) Lateral Meniscal Height (LMW) Lateral Meniscal Width (LM-LEW) LM Lateral Eminence Width.

**Fig 2:**
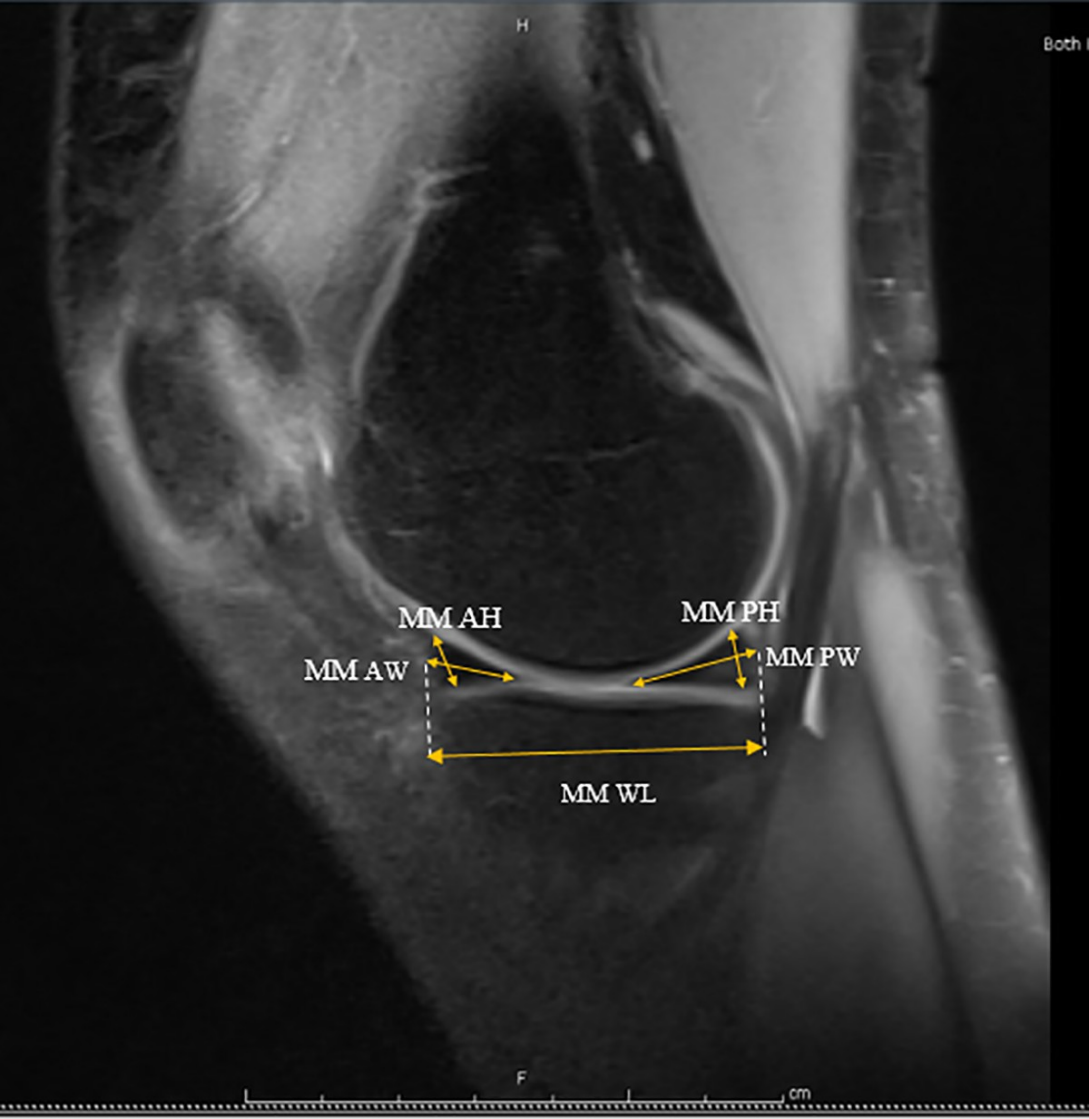
Mid-medial sagittal section (MM AH) Medial Meniscal Anterior Height (MM AW) Medial Meniscal Anterior Width (MM PH) Medial Meniscal Posterior Height (MM PW) Medical Meniscal Posterior Width (MM WL) Medial Meniscal Whole Length.

**Fig 3:**
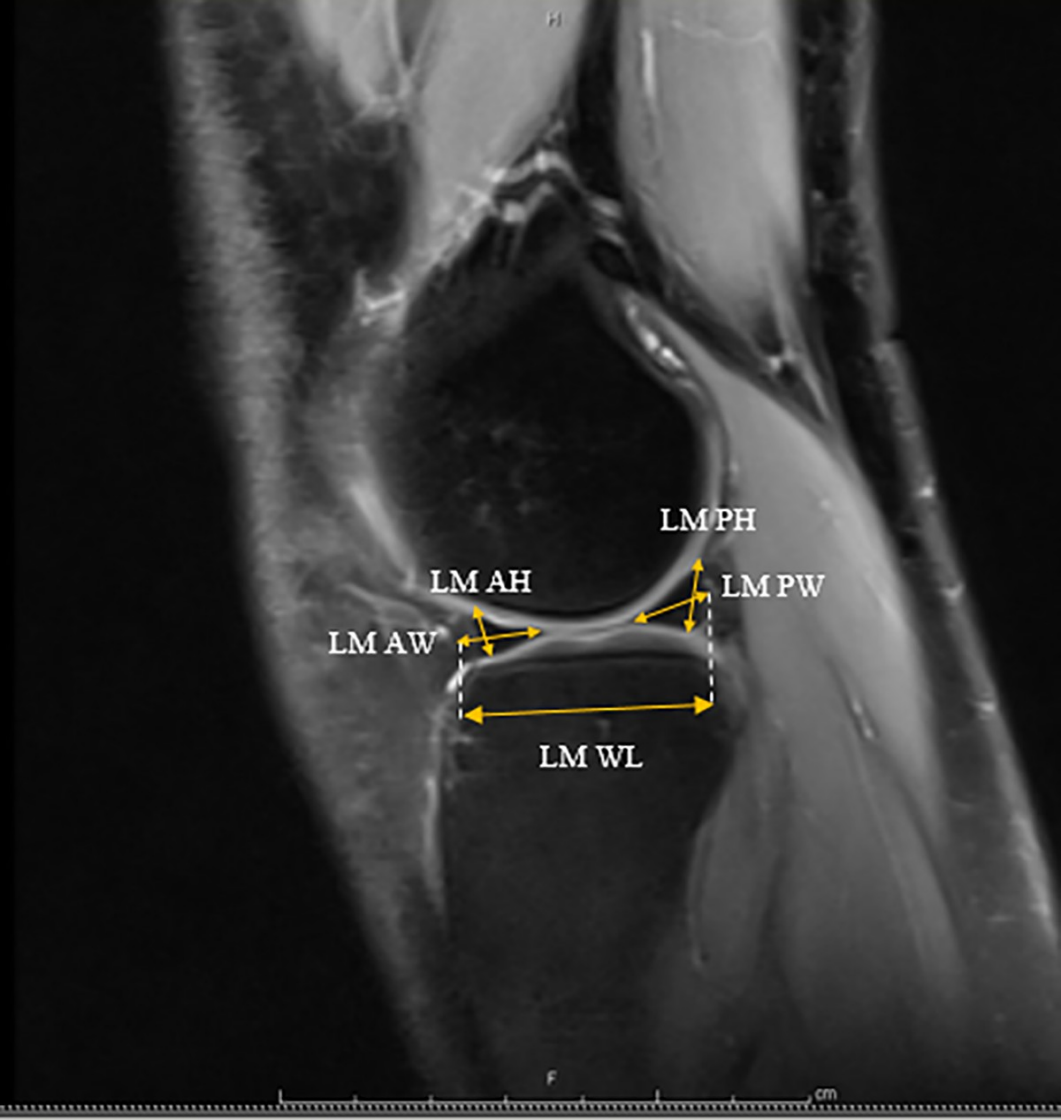
Mid-lateral sagittal section (LM AH) Lateral Meniscal Anterior height, (LM AW) Lateral Meniscal Anterior Width (LM PH) Lateral Meniscal Posterior Height (LM PW) Lateral Meniscal Posterior Width (LM WL) Lateral Meniscal Whole Length.

Each individual was examined with knee joints in full extension. By using mid-coronal images, the medial meniscus and lateral meniscus length, width, and lateral eminence were measured (Fig 1). The distances from the medial eminence to the meniscocapsular junction of the MM and from the lateral eminence to the meniscocapsular junction of the LM were also measured. Mid-sagittal images were used to measure the height and width of the anterior and posterior horns of each meniscus, as well as the whole length spanning from the most anterior margin of each meniscus to the most posterior (Figs 2 & 3). Measurements were performed two separate times, two weeks apart. A mean of three measurements was made during each session to reduce error.

### Inclusion and exclusion criteria

Only patients who had normal bilateral knee MRIs were selected. Individuals under 18 and above 50 years were excluded from the study, in addition to those who had a history of meniscal tear or repair. Patients with pathologies that were suspected to reduce the accuracy of these measurements were also excluded. Such pathologies included a discoid meniscus, fractures of the lower limb, rheumatoid arthritis, trauma of the knee with associated bone bruising, osteoarthritis of the knee, and signs of degeneration.

Thirty-eight normal bilateral knee joints MRIs remained (16 males, 22 females). Participants were sampled from the institutional Picture Archiving and Communication System (PACS). Age, gender, and medial meniscal and lateral meniscal size of both knees were recorded. The laterality of the menisci was compared between both knees in each patient.

### Statistical analysis

SPSS version 21.0 (Chicago, USA) was used in our analysis. The mean (± standard deviation) was utilized to describe continuous variables (i.e. age and measurements). Count (frequency) was utilized to describe other nominal variables (i.e. gender). Paired-sample t-test was used to compare right and left meniscal measurements. An independent sample t-test was performed to analyze the mean difference between measurements and gender, and data were presented in mean (95% confidence interval (CI)). The correlation between each measurement with age was determined using Pearson’s correlation coefficient. All underlying assumptions were met unless otherwise indicated. We adopted a p-value of 0.05 as a significant threshold.

## Results

A total of 38 patients were included in this study, with a mean age of 37.39 (±9.50) years. They were 16 (42.1%) men and 22 (57.9%) women.

We didn’t find any significant difference in the mid-coronal section between left and right knees meniscal measurements. The mean difference in measurements ranged from as low as 0.009 (95% CI -0.38 to 0.36) in MMH to as high as 0.44 (95% CI -0.59 to 0.50) in LMW. Table 1 details the measurements in the mid-coronal view. None of the measurements correlated with age or gender.

**Table 1:** Mean differences and the significance of the differences between left and right knee meniscal measurements in mid-coronal view. The minus sign here means that the left was more than the right.

		Mean	Standard Deviation	Mean difference	95% Confidence interval		P value
					Lower	Upper	
Pair 1	Right MMH	5.0371	1.12324	-0.009	-0.38	0.36	0.96
	Left MMH	5.0461	.90842				
Pair 2	Right MMW	8.4032	2.60581	0.54	-0.48	1.56	0.29
	Left MMW	7.8658	2.41042				
Pair 3	Right MM-MEW	28.5524	4.57837	-0.60	-1.49	0.29	0.18
	Left MM-MEW	29.1539	3.99853				
Pair 4	Right LMH	5.6116	1.22198	-0.12	-0.99	0.33	0.33
	Left LMH	5.7316	.93774				
Pair 5	Right LMW	9.7968	2.89690	0.10	-0.45	0.66	0.73
	Left LMW	9.6992	2.85743				
Pair 6	Right LM-LEW	30.2611	3.83217	0.23	-0.45	0.90	0.50
	Left LM-LEW	30.0355	3.87666				

MMH: Medial Meniscal Height; MMW: Medial Meniscal Width; MM-MEW: Medial Meniscal Medial Eminence Width; LMH: Lateral Meniscal Height; LMW: Lateral Meniscal Width; LM-LEW: Lateral Meniscal Lateral Eminence Width

There was no significant difference in the medial mid-sagittal section between left and right knee meniscal measurements. The mean difference in measurements ranged from as low as 0.04 (95% CI -0.46 to 0.39) in posterior MMH to as high as 0.39 (95% CI -0.07 to 0.85) in anterior Medial Meniscal Width (MMW). Table 2 details the measurements in the lateral mid-sagittal view. We found that age significantly (p = 0.043) correlated with left posterior MMW with a negative correlation coefficient of -0.303. None of the measurements were significantly different between men and women.

**Table 2:** Mean differences and the significance of the differences between the left and right knee meniscal measurements in the medial mid-sagittal view.

		Mean	Standard Deviation	Mean difference	95% Confidence interval		P value
					Lower	Upper	
Pair 1	Right Anterior MMH	4.9655	1.04964	0.24	-0.09	0.56	0.17
	Left Anterior MMH	4.7297	1.12912				
Pair 2	Right Anterior MMW	8.6745	1.26929	0.39	-0.07	0.85	0.09
	Left Anterior MMW	8.2837	1.77826				
Pair 3	Right Posterior MMH	6.0374	1.33294	-0.04	-0.46	0.39	0.89
	Left Posterior MMH	6.0726	1.56879				
Pair 4	Right Posterior MMW	13.7434	3.39383	-0.11	-0.96	0.74	0.80
	Left Posterior MMW	13.8518	3.57912				
Pair 5	Right MM Whole length	41.7797	4.71250	-0.25	-1.29	0.78	0.62
	Left MM Whole length	42.0345	4.90259				

MMH: Medial Meniscal Height; MMW: Medial Meniscal Width

There was no significant difference in the lateral mid-sagittal section between left and right knee meniscal measurements. The mean difference in measurements ranged from as low as 0.13 (95% CI -0.62 to 0.35) in posterior Lateral Meniscal Height (LMH) to as high as 0.96 (95% CI -0.20 to 2.13) in Lateral Meniscal (LH) whole length. Table 3 details the measurements in the lateral mid-sagittal view. We found that men have significantly (p = 0.011) higher right LM anterior height compared to women with a mean height difference of 0.52 (95% CI -0.27 to 1.31). Men have also had significantly (p = 0.014) higher right LM anterior width compared to women with a mean width difference of 1.86 (95% CI 0.80 to 2.91). Age did not correlate with any of the measurements.

**Table 3:** Mean differences and the significance of the differences between left and right knee meniscal measurements in the lateral mid-sagittal view.

		Mean	Standard Deviation	Mean difference	95% Confidence interval		P value
					Lower	Upper	
Pair 1	Right Anterior LMH	4.7321	1.33722	-0.18	-0.51	0.15	0.31
	Left Anterior LMH	4.9126	1.43220				
Pair 2	Right Anterior LMW	9.4587	1.96066	-0.13	-0.62	0.35	0.46
	Left Anterior LMW	9.5929	2.10319				
Pair 3	Right Posterior LMH	6.2174	1.57665	0.15	-0.27	0.58	0.64
	Left Posterior LMH	6.0655	1.19097				
Pair 4	Right Posterior LMW	9.6011	2.40458	0.36	-0.53	0.60	0.86
	Left Posterior LMW	9.5647	1.72314				
Pair 5	Right LM Whole Length	34.1937	6.00927	0.96	-0.20	2.13	0.13
	Left LM Whole Length	33.2308	4.93239				

LMH: Lateral Meniscal Height; LMW: Lateral Meniscal Width; LM: Lateral Meniscal

## Discussion

MAT surgery is becoming the preferred treatment for symptomatic patients with a partial or total loss of the meniscus. These procedures are utilized to delay the early development of knee degenerative changes after total or partial meniscectomies. The classic indications for these surgeries include the absence of advanced degenerative changes in the knee and the correction of any concomitant pathology such as malalignment, localized osteochondral defect, or instability. However, these indications are being expanded to include patients with more prominent degenerative changes after studies have shown that 73.5% of patients can resume sporting activities postoperatively. There is, however, a 22.4% failure rate for the MATs [5].

Failure of meniscal allograft surgery is commonly caused by extrusion or tearing. Extrusion may become more likely if the menisci are oversized. On the other hand, an undersized meniscal allograft may result in tears, as a result of increased contact pressures [6]. Therefore, the importance of having an accurate way to measure meniscal allografts is to preserve function and reduce failure rates of MAT.

To determine the sizing of a meniscal allograft, preoperative imaging measuring the bony landmarks and points of insertion of soft tissue can be used. The standard method for determining the MAT size is the method developed by Pollard et al depending on x-ray measurements with a reported mean error of estimating the size of the native meniscus of less than 8.4% [7]. However, to determine the size of the allograft using this method, preoperative imaging measuring the bony landmarks and points of insertion of soft tissue are used. This method, however, may yield inaccuracies as a result of errors in magnification and incorrect identification of landmarks [5]. Studies have suggested that to preserve the anatomic and biochemical limitations of the original meniscus, the size should be accurate to at least 5mm [8]. Another study proposed a 10% limitation for the difference between the original meniscus and the allograft [9]. This limitation of size difference and the need for more accurate sizing of the allograft may justify the use of this more costly method of imaging, compared to the use of x-ray imaging. In addition, variations in points of insertion of soft tissue can be better viewed using MRI.

In our study, in the mid-coronal view, the greatest difference in size between the right and left menisci was 0.44 mm. When noting the mean differences between the left and right knee meniscal measurements in the medial mid-sagittal view, the mean differences between the menisci in both knees ranged from 0.04 to 0.39 mm. In the lateral mid-sagittal view, the differences in size ranged from 0.13 to 0.96 mm. The p-values for all these measurements were all over 0.05, with a confidence interval of 95%. These results correspond to those found in similar studies [9] [10] [11] [12]. The results obtained in this study support the use of MRI of the contralateral knee to obtain an appropriately sized allograft.

Of the limitations of this study was the sample size of 38 participants. If this study included a larger sample size, then the significance of the study could be enhanced. In addition, using contralateral knee MRI measurements in a knee with pathology may not be a useful or accurate method of determining meniscal size. Besides, the use of MRI measurements carries a much higher cost than the use of x-ray imaging, which may outweigh the potential benefits of this method. However, this is one of few studies studying the use of these measurements in the Middle Eastern patient population.

## Conclusion

The use of MRI to determine the size of contralateral meniscus was shown to be an accurate method of measurement. Due to the fact that failure of MAT is usually due to errors in sizing, this method may significantly improve the outcomes of this surgery, and therefore, justify the higher cost of this modality of imaging.

## Supporting information

S1 File MRI Measurements of the Knee. The measurements taken from the MRIs of the knees of healthy patients in cm.
(XLSX)
